# Liposomal irinotecan with fluorouracil and leucovorin as salvage treatment for advanced biliary tract cancer refractory to gemcitabine and cisplatin

**DOI:** 10.3389/fonc.2025.1638606

**Published:** 2025-08-13

**Authors:** Hyunho Kim, Kabsoo Shin, Hyung Soon Park, Tae Ho Hong, Younghoon Kim, Sung Hak Lee, In-Ho Kim, MyungAh Lee, Se Jun Park

**Affiliations:** ^1^ Division of Medical Oncology, Department of Internal Medicine, St. Vincent’s Hospital, College of Medicine, The Catholic University of Korea, Seoul, Republic of Korea; ^2^ Division of Medical Oncology, Department of Internal Medicine, Seoul St. Mary’s Hospital, College of Medicine, The Catholic University of Korea, Seoul, Republic of Korea; ^3^ Cancer Research Institute, College of Medicine, The Catholic University of Korea, Seoul, Republic of Korea; ^4^ Department of General Surgery, Seoul St. Mary’s Hospital, College of Medicine, The Catholic University of Korea, Seoul, Republic of Korea; ^5^ Department of Pathology, College of Medicine, The Catholic University of Korea, Seoul St. Mary’s Hospital, Seoul, Republic of Korea

**Keywords:** biliary tract cancer, liposomal irinotecan, second-line chemotherapy, real-world evidence, survival outcome

## Abstract

**Introduction:**

The combination of liposomal irinotecan with fluorouracil and leucovorin (Nal-IRI/FL) has shown efficacy in phase II trials for advanced biliary tract cancer (BTC) following gemcitabine-cisplatin (GP) therapy. However, its effectiveness and safety in real-world clinical settings have not been well established. This study aimed to assess the real-world outcomes of Nal-IRI/FL in BTC patients who experienced disease progression after gemcitabine-based treatment.

**Materials and methods:**

This retrospective, multicenter study evaluated patients with advanced BTC who received Nal-IRI/FL following progression on GP-based therapy between January 2022 and December 2024. Survival outcomes, radiologic responses, toxicities, and molecular alterations were evaluated, with key findings compared against those reported in prior clinical trials.

**Results:**

A total of 93 patients were included. The median progression-free survival (PFS) and overall survival (OS) were 2.1 and 4.2 months, respectively. Among 76 radiologic response evaluable patients, median PFS and OS were 2.5 and 5.0 months. The disease control rate was 40.8%, and objective response rate was 7.5%. Higher disease burden and poor performance status were associated with inferior outcomes. Efficacy did not significantly differ between second- and third-line settings or based on *RAS* or *TP53* mutation status. Hematological toxicities were common, including grade ≥3 neutropenia (38.7%) and febrile neutropenia (7.5%). The median relative dose intensity was 0.69. Treatment-related death occurred in 4 patients (4.3%).

**Conclusions:**

Nal-IRI/FL showed modest effectiveness in real-world settings, with outcomes generally less favorable than clinical trials, potentially reflecting patient characteristics. Its efficacy was consistent across treatment lines and mutation subgroups, including patients with *RAS* or *TP53* mutations. Careful patient selection and proactive supportive care are essential. Further studies are warranted to clarify its role across diverse populations.

## Introduction

Biliary tract cancers (BTCs), encompassing intrahepatic and extrahepatic cholangiocarcinoma as well as gallbladder cancer, are highly lethal malignancies frequently diagnosed at advanced stages, with poor prognosis when curative surgery is not feasible (1). Even after curative resection, recurrence rates remain high, with a 5-year survival rate of only 25%, resulting in the majority of patients with BTCs ultimately requiring systemic therapy (2, 3). The combination of gemcitabine and cisplatin (GP) has served as the cornerstone of first-line treatment in advanced BTCs for over a decade (4). Recent phase III studies have demonstrated that the addition of immune checkpoint inhibitors, such as durvalumab or pembrolizumab, to GP significantly improves overall survival (OS) compared to chemotherapy alone, with median survival now exceeding one year (5, 6).

Following progression on first-line GP-based therapy, treatment options have historically been limited. While targeted therapies may be effective for patients with advanced BTC harboring actionable genetic alterations such as *ERBB2* amplification, *FGFR2* fusions or rearrangements, or *IDH1* mutations, treatment options remain limited for those without such alterations (7). In the absence of druggable molecular alterations, fluorouracil-based chemotherapy may be considered as a second-line treatment. The phase III ABC-06 trial supported the use of FOLFOX (fluorouracil, leucovorin, and oxaliplatin) as a second-line option, although the OS advantage relative to active symptom control was modest (median OS, 6.2 months [95% CI, 5.4–7.6] vs. 5.3 months [4.1–5.8]; hazard ratio [HR], 0.69 [0.50–0.97]; *p* = 0.031) (8).

Additionally, the phase II NIFTY trial demonstrated that the combination of liposomal irinotecan with fluorouracil and leucovorin (Nal-IRI/FL) significantly improved progression-free survival (PFS) compared to FL alone in patients with advanced BTC who had progressed on GP (median PFS, 7.1 months [95% CI, 3.6–8.8] vs. 1.4 months [1.2–1.5]; HR, 0.56 [0.39–0.81]; *p* = 0.0019) (9), suggesting that Nal-IRI/FL may represent a viable alternative option. However, an updated analysis of the NIFTY trial showed that although the median PFS in the Nal-IRI/FL group remained significantly superior to that of the FL group (4.2 months [95% CI, 2.8–5.3] vs. 1.7 months [1.4–2.6]; HR, 0.61 [0.44–0.86]; *p* = 0.004), it was numerically lower than in the initial report (10). Moreover, the NALIRICC phase II trial conducted in a German population found that the addition of liposomal irinotecan to FL did not improve PFS (2.6 months [95% CI, 1.7–3.6] vs. 2.3 months [1.6–3.4]; HR, 0.87 [0.56–1.35]; *p* = 0.52) and was associated with increased toxicity compared to FL alone (11). These inconsistent findings suggest that the role of Nal-IRI/FL as a second-line treatment in advanced BTC remains uncertain and warrants further investigation.

Given the inconsistent results from previous clinical trials and the limited evidence available for broader patient populations, further investigation of the Nal-IRI/FL regimen in routine clinical practice is warranted. Real-world data can offer meaningful insights into its clinical effectiveness and safety, particularly in patients who may not meet strict eligibility criteria for prospective trials. In light of these considerations, we conducted a retrospective, multicenter analysis to evaluate the real-world outcomes of Nal-IRI/FL administered as second or later-line therapy in patients with advanced BTC following progression on GP-based therapy, with particular focus on treatment effectiveness and tolerability.

## Materials and methods

### Patients

This retrospective study included patients with histologically or cytologically confirmed advanced BTC who experienced disease progression following GP-based chemotherapy, including neoadjuvant, adjuvant, or palliative settings. Medical records were reviewed from Seoul St. Mary’s Hospital and St. Vincent’s Hospital. The study was conducted in accordance with Korean regulations and the Declaration of Helsinki, and was approved by the Institutional Review Board (IRB) of The Catholic University of Korea, Seoul St. Mary’s Hospital (approval ID: KC25RASI0352). The ethics committee waived the requirement for written informed consent due to the retrospective nature of the study.

### Procedures

Patients received the Nal-IRI/FL regimen, which consisted of intravenous liposomal irinotecan 70 mg/m² administered over 90 minutes, followed by leucovorin 400 mg/m² intravenously over 30 minutes, and fluorouracil 2400 mg/m² as a continuous intravenous infusion over 46 hours, repeated every 2 weeks. Treatment was continued until radiological or clinical disease progression, as assessed by the treating physician, or the occurrence of unacceptable toxicity. Dose and schedule modifications were permitted at the physician’s discretion to manage adverse events.

Relative dose intensity (RDI) was defined as the ratio of the delivered dose intensity to the planned dose intensity over a specified period, expressed as a percentage. RDI was evaluated in patients who received at least two cycles of chemotherapy.

### Assessments

Tumor response was evaluated based on the Response Evaluation Criteria in Solid Tumors (RECIST) version 1.1. Imaging assessments, including computed tomography or magnetic resonance imaging of the chest, abdomen, and pelvis, were performed every 6 to 8 weeks after treatment initiation. Additional imaging studies were conducted when clinically indicated. Serum carbohydrate antigen 19-9 (CA19-9) levels were assessed concurrently with radiologic evaluations. Adverse events were assessed at each clinical encounter and graded according to the National Cancer Institute Common Terminology Criteria for Adverse Events (CTCAE), version 5.0. At each visit, patients were assessed with physical examination, Eastern Cooperative Oncology Group (ECOG) performance status, symptom review, and laboratory tests. Patients were considered evaluable for radiologic response if they had received at least one cycle of treatment and had undergone at least one post-baseline imaging assessment. Fluorouracil-refractory disease was defined as progression occurring during fluorouracil-based chemotherapy or within 3 months of its discontinuation. Fluorouracil-resistant disease was defined as progression occurring between 3 and 6 months after completing such therapy.

### Molecular alteration analyses

In a subset of patients, molecular profiling was performed using formalin-fixed, paraffin-embedded tumor tissue obtained either at the time of diagnosis or upon disease recurrence. Next-generation sequencing (NGS) was conducted using the Oncomine™ Comprehensive Assay Plus (Thermo Fisher Scientific, Waltham, MA, USA), which analyzes 517 cancer-related genes, including oncogenes, tumor suppressor genes, gene fusions, microsatellite instability status, and tumor mutational burden. The therapeutic outcomes were compared according to the presence or absence of specific molecular alterations, including *RAS* and *TP53* mutations.

### Statistical analysis

Descriptive statistics are presented as proportions for categorical variables and medians with interquartile ranges (IQR) for continuous variables. Categorical variables were compared using the chi-square test or Fisher’s exact test, as appropriate. Continuous variables such as age were not statistically compared, as only summary-level data were available from previously published clinical trials. Fisher’s exact test was used for pairwise comparisons of objective response rate (ORR) and disease control rate (DCR) between the current study and prior prospective trials. OS was defined as the time from initiation of chemotherapy to death from any cause. PFS was defined as the time from the initiation of chemotherapy to either radiographic disease progression or death, whichever occurred first. Kaplan–Meier methodology was used to generate non-parametric estimates of median OS and PFS. Hazard ratios (HRs) and 95% confidence intervals (CIs) were estimated using unstratified Cox proportional hazards regression to assess the effects of treatment and other potential prognostic variables on survival outcomes. Variables considered for univariable analysis were selected based on clinical relevance and potential association with survival, while those included in multivariable analysis were limited to variables that showed statistical significance in univariable analysis or were recognized as established prognostic factors. All statistical tests were two-sided, and *p*-values less than 0.05 were considered statistically significant. Statistical analyses were performed using SPSS for Windows, version 24.0 (IBM Corp., Armonk, NY, USA), and GraphPad Prism, version 10.3 (GraphPad Software, San Diego, CA, USA).

## Results

### Patients

**Figure 1:**
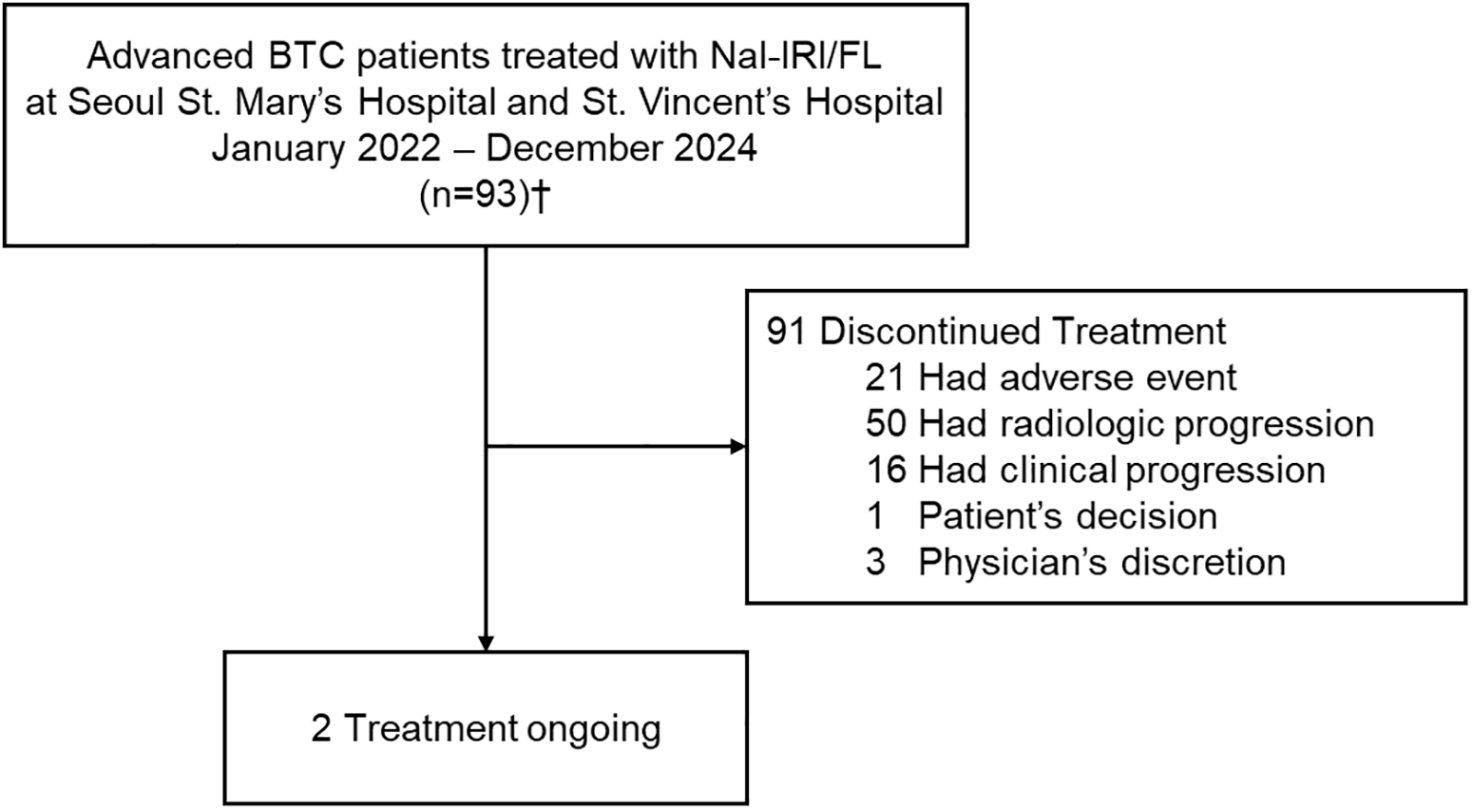
Flow diagram of patients in real-world analysis. †Of the 93 patients who received Nal-IRI/FL, 76 were evaluable for radiologic response. BTC, biliary tract cancer; Nal-IRI/FL, fluorouracil, leucovorin and liposomal irinotecan.

Between January 2022 and December 2024, a total of 93 patients with advanced BTCs who received Nal-IRI/FL as second- or third-line therapy were retrospectively identified and included in the analysis. Of these, treatment was discontinued in 91 patients, while 2 were still receiving Nal-IRI/FL at the time of data cutoff (**Figure 1**). Baseline characteristics of this cohort were compared with those reported in the NIFTY and NALIRICC trials (**Table 1**). The median age was 65 years (IQR, 57–72), comparable across all three cohorts. Although gender distribution showed a statistically significant difference (*p* = 0.042), the overall proportions of male and female patients were broadly similar. A significantly higher proportion of patients with ECOG performance status 2 was observed in our cohort (*p* < 0.001). The distribution of primary tumor sites also differed significantly (*p* = 0.004), with intrahepatic cholangiocarcinoma being predominant in NALIRICC, whereas our cohort and NIFTY exhibited a more balanced distribution across intrahepatic, extrahepatic, and gallbladder cancers. Prior curative surgery was more commonly reported in our study (54.8%) and NALIRICC (61.2%) compared to NIFTY (29.5%, *p* = 0.001). Regarding treatment history, approximately one-quarter of patients in our cohort received Nal-IRI/FL as third-line therapy (25.8%, *p* < 0.001).

**Table 1:** Baseline characteristics.

Variable	Present study (n=93)	NIFTY (n=88)	NALIRICC (n=49)	*p* value*
Age, years	65 (57-72)	63 (38-84)	66 (59-70)	
Gender				
Female	39 (41.9)	37 (42.0)	23 (46.9)	0.042
Male	54 (58.1)	51 (58.0)	26 (53.1)	
ECOG performance status				
0	21 (22.6)	23 (26.1)	33 (67.3)	<0.001
1	53 (57.0)	65 (73.9)	16 (32.7)	
2	19 (20.4)	0	0	
Disease stage				
Locally advanced	2 (2.2)	0	2 (4.0)	
Metastatic	91 (97.8)	88 (100)	47 (96.0)	
Primary tumor location				
Intrahepatic	28 (30.1)	35 (39.8)	30 (61.2)	0.004
Extrahepatic	37 (39.8)	22 (25.0)	10 (20.4)	
Gallbladder	28 (30.1)	31 (35.2)	9 (18.4)	
Had previous surgery	51 (54.8)	26 (29.5)	30 (61.2)	0.001
Previous gemcitabine and cisplatin				
Duration, months	4.9 (3.0-6.5)	5.1 (3.0-7.0)	4.2 (NR)	
≥ 6 months	27 (29.0)	31 (30.4)	NR	
Prior lines of therapy†				
1	69 (74.2)	88 (100)	44 (89.8)	<0.001
≥ 2	24 (25.8)	0	5 (10.2)	
Fluorouracil sensitivity				
Resistant or refractory	26 (27.9)	NR	NR	
Site of metastasis				
Liver	59 (63.4)	59 (67.0)	34 (69.4)	
Lung	29 (31.2)	22 (25.0)	17 (34.7)	
Lymph node	50 (53.8)	57 (64.8)	24 (49.0)	
Peritoneum	34 (36.5)	25 (28.4)	11 (22.4)	
Bone	10 (10.7)	5 (5.7)	8 (16.3)	
Baseline CA 19-9				
UmL	31 (33.3)	48 (54.5)	NR	0.005
≥172 U/mL	62 (66.7)	40 (45.5)	NR	

ECOG, Eastern Cooperative Oncology Group; NR, not reported; CA 19–9, carbohydrate antigen 19-9. Data are n (%) or median (IQR). *Statistical comparisons for continuous variables were not performed owing to the unavailability of individual-level data. †Systemic treatment for metastatic disease, including cytotoxic chemotherapy, targeted therapy, and immune checkpoint inhibitors.

### Effectiveness

**Figure 2:**
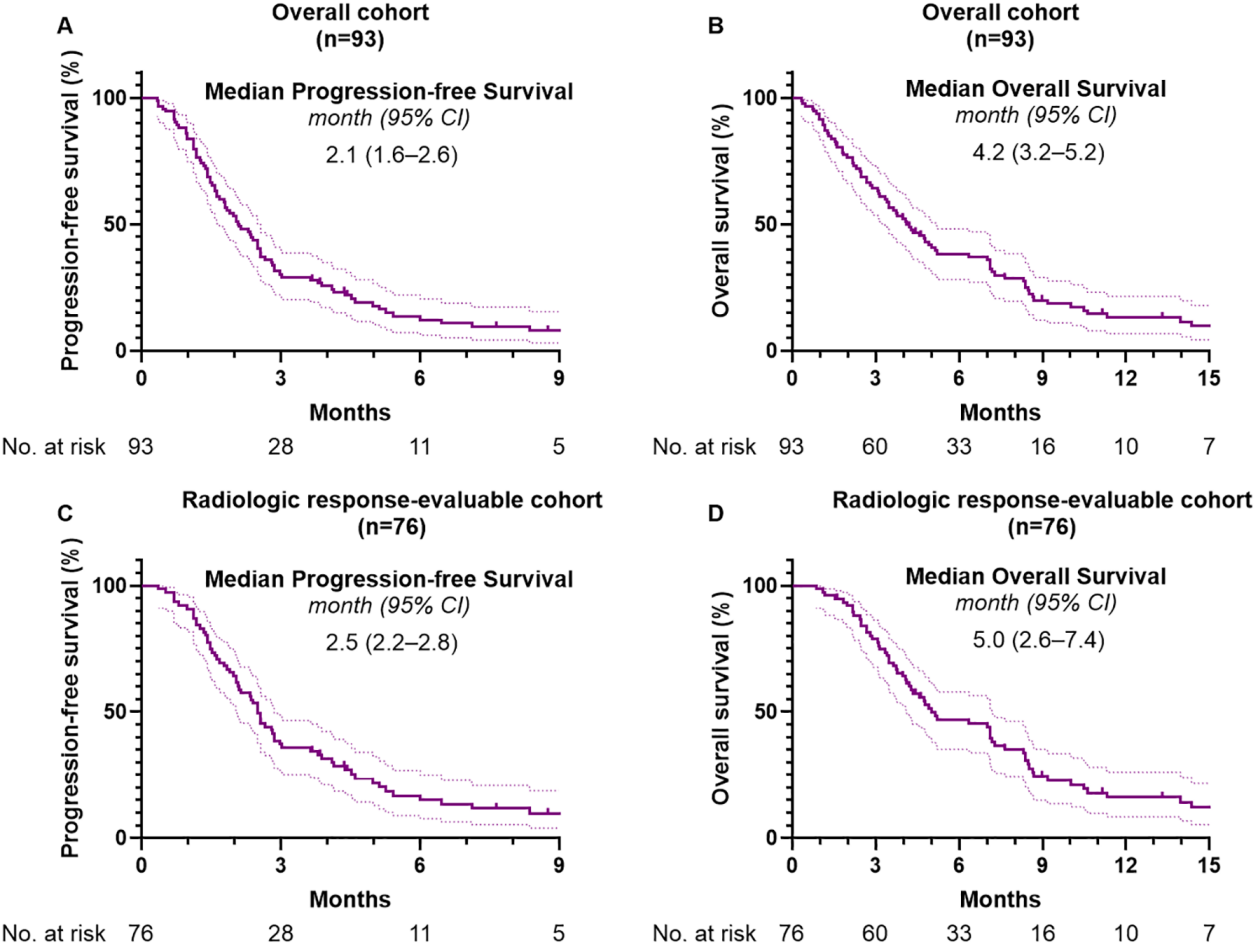
Kaplan-Meier analysis of survival outcomes. **(A)** Progression-free survival and **(B)** overall survival in the overall cohort. **(C)** Progression-free survival and **(D)** overall survival in the radiologic response-evaluable subgroup.

The median follow-up duration was 4.1 months (95% CI, 3.2–4.8). Among the 93 patients included in this study, 85 (91.4%) experienced disease progression and 83 (89.2%) had died at the time of analysis. The median PFS and OS in the overall cohort were 2.1 months (95% CI, 1.6–2.6) and 4.2 months (95% CI, 3.2–5.2), respectively (**Figures 2A, B**). A In the subgroup of 76 patients (81.7%) evaluable for radiologic response, median PFS was 2.5 months (95% CI, 2.2–2.8) and median OS was 5.0 months (95% CI, 2.6–7.4) (**Figures 2C, D**). **Table 2** summarizes the effectiveness outcomes in comparison with the NIFTY and NALIRICC trials. The median PFS in our cohort was shorter than that observed in NIFTY (4.2 months) and comparable to NALIRICC (2.6 months). The 6-month PFS rate was 13.7%, compared to 31.8% in NIFTY and 23.0% in NALIRICC. Similarly, the median OS in our cohort was 4.2 months, whereas it was 8.6 months in NIFTY and 6.9 months in NALIRICC. The 6-month OS rate was 38.3%, compared to 60.7% in NIFTY and 50.0% in NALIRICC. The ORR was 7.5%, which was numerically lower than that reported in NIFTY (14.8%) and NALIRICC (14.3%), though not statistically significant (*p* = 0.156 and *p* = 0.240, respectively). The DCR was 40.8%, which was significantly lower than that of NIFTY (64.8%, *p* = 0.018) but not significantly different from NALIRICC (51.0%, *p* = 0.290).

**Table 2:** Clinical outcomes of liposomal irinotecan with fluorouracil, leucovorin in patients with advanced biliary tract cancer.

Variable	Present study	NIFTY	NALIRICC
	(n=93)	(n=88)	(n=49)
Best overall response, n (%)			
Partial response	7 (7.5)	13 (14.8)	7 (14.3)
Stable disease	31 (33.3)	44 (50.0)	18 (36.7)
Progressive disease	38 (40.9)	26 (29.5)	12 (24.5)
Not evaluable	17 (18.3)	5 (5.7)	12 (24.5)
Objective response rate, n (%)	7 (7.5)	13 (14.8)	7 (14.3)
*P* value vs. Present study		0.156	0.24
Disease control rate, n (%)	38 (40.8)	57 (64.8)	25 (51.0)
*P* value vs. Present study		0.018	0.29
Median PFS, months [95% CI]	2.1 [1.6–2.6]	4.2 [2.8–5.3]	2.6 [1.7–3.6]
6-month PFS, % [95% CI]	13.7 [7.3–22.1]	31.8 [21.7–41.8]	23.0 [10.0–35.0]
Median OS, months [95% CI]	4.2 [3.2–5.2]	8.6 [5.4–10.5]	6.9 [5.3–10.6]
6-month OS, % [95% CI]	38.3 [28.3–48.2]	60.7 [50.3–71.2]	50.0 [40.0–70.0]

PFS, progression-free survival; OS, overall survival.

### Multivariable analysis for survival outcomes

**Table 3** summarizes the results of univariable and multivariable analyses evaluating potential prognostic factors for survival outcomes. In the multivariable model for PFS, having three or more metastatic lesions was the only variable significantly associated with poorer PFS (HR = 1.86; 95% CI, 1.16–2.99; *p* = 0.010). Other clinical factors, including number of prior treatment lines and fluorouracil resistance, were not significantly associated with PFS. Similarly, in the multivariable analysis for OS, the presence of three or more metastatic lesions was independently associated with inferior OS (HR = 2.39; 95% CI, 1.49–3.83; *p* = 0.001), and poor performance status was also significantly associated with worse outcomes (HR = 1.81; 95% CI, 1.00–3.27; *p* = 0.049). Moreover, a history of prior curative surgery showed a trend toward improved OS, although it did not reach statistical significance (HR = 0.65; 95% CI, 0.40–1.05; *p* = 0.080).

**Table 3:** Univariable and multivariable analyses of clinicopathological factors potentially associated with progression-free survival and overall survival.

Variables	PFS				OS			
	Univariable analysis		Multivariable analysis		Univariable analysis		Multivariable analysis	
	HR (95% CI)	*p* value	HR (95% CI)	*p* value	HR (95% CI)	*p* value	HR (95% CI)	*p* value
Age ≥65 (vs. <65 year)	0.91 (0.59–1.39)	0.658			1.36 (0.87–2.15)	0.179		
Female (vs. male)	0.84 (0.54–1.30)	0.434			0.72 (0.47–1.13)	0.157		
ECOG PS 2 (vs. PS 0–1)	1.17 (0.69–1.98)	0.566	1.15 (0.63–2.07)	0.654	1.72 (1.02–2.89)	0.042	1.81 (1.00–3.27)	0.049
Primary tumor location								
EHCC (vs. IHCC)	0.88 (0.52–1.49)	0.635			1.25 (0.73–2.15)	0.419		
GBC (vs. IHCC)	1.03 (0.59–1.80)	0.915			1.45 (0.83–2.51)	0.188		
No. of metastatic lesion ≥3 (vs. <3)	1.81 (1.15–2.86)	0.011	1.86 (1.16–2.99)	0.010	2.02 (1.29–3.17)	0.002	2.39 (1.49–3.83)	0.001
Previous surgery (vs. none)	0.66 (0.42–1.03)	0.066	0.69 (0.42–1.11)	0.127	0.59 (0.38–0.91)	0.018	0.65 (0.40–1.05)	0.080
Previous GP ≥6 months (vs. < 6 months)	1.10 (0.68–1.77)	0.694			0.67 (0.41–1.09)	0.105		
Prior lines of therapy ≥2 (vs. <2)	1.25 (0.76–2.06)	0.375			1.24 (0.74–2.07)	0.404		
Fluorouracil resistance (vs. none)	1.12 (0.70–1.80)	0.637			1.19 (0.73–1.92)	0.489		
CA 19-9 ≥172 U/mL (vs. <172 U/mL)	1.05 (0.66–1.67)	0.829			1.30 (0.82–2.05)	0.266		

PFS, progression-free survival; OS, overall survival; HR, hazard ratio; ECOG PS, Eastern Cooperative Oncology Group Performance Status; IHCC, intrahepatic cholangiocarcinoma; EHCC, extrahepatic cholangiocarcinoma; GBC, gallbladder cancer; GP, gemcitabine with cisplatin; CA 19–9, carbohydrate antigen 19-9.

### Survival outcomes by mutational status

**Figure 3:**
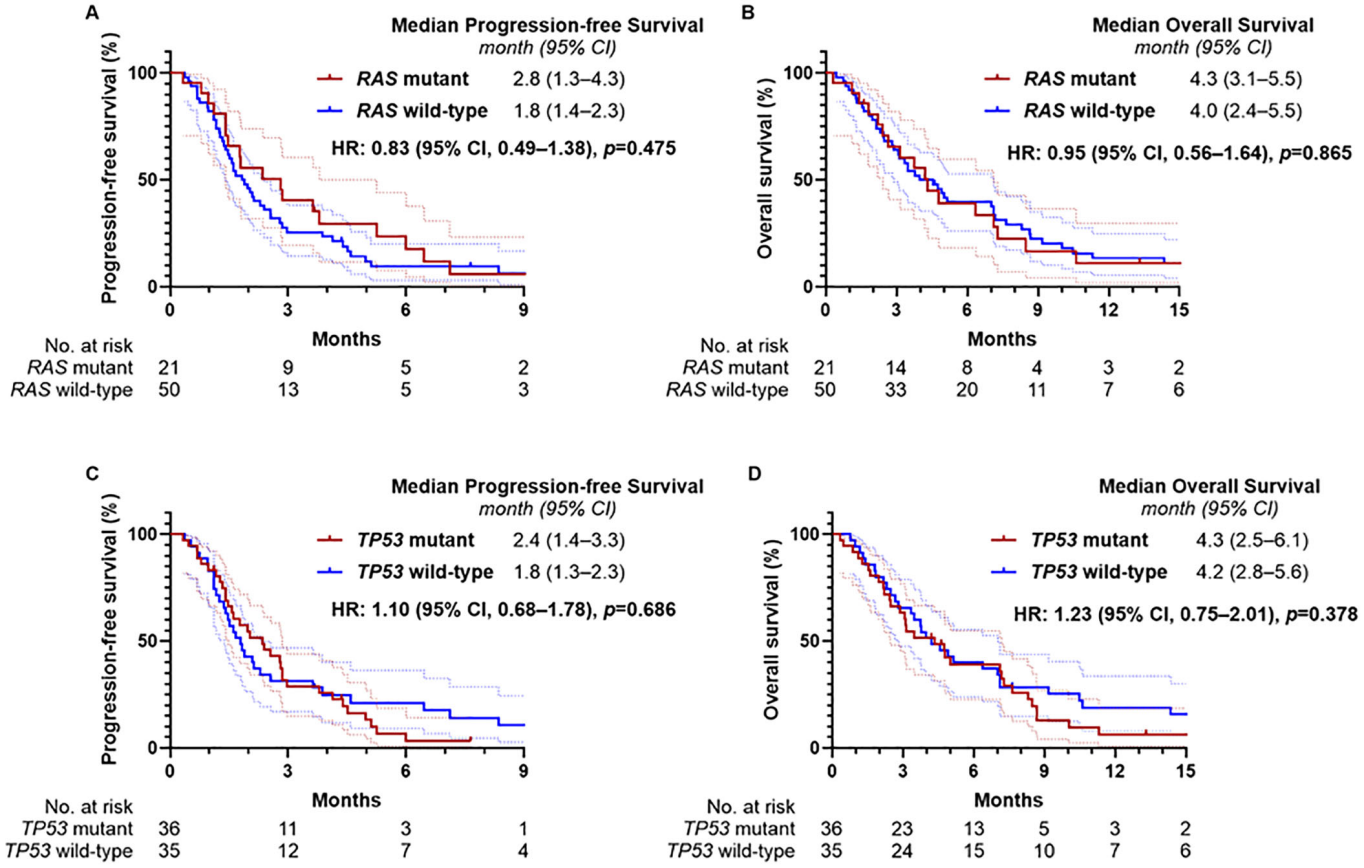
Kaplan-Meier estimates of progression-free survival and overall survival according to *RAS* and *TP53* mutation status. **(A)** Progression-free survival and **(B)** overall survival according to RAS mutation status, and **(C)** progression-free survival and **(D)** overall survival according to TP53 mutation status.

Tissue-based NGS was performed in 71 of the 93 patients (76.3%), and molecular profiling results are summarized in **Supplementary Table S1**. Among these, *RAS* mutations were identified in 21 patients (29.5%) and *TP53* mutations in 36 (50.7%). Median PFS was 2.8 months (95% CI, 1.3–4.3) in the *RAS*-mutant group and 1.8 months (95% CI, 1.4–2.3) in the wild-type group (*p* = 0.475, **Figure 3A**). Median OS was 4.3 months (95% CI, 3.1–5.5) in the *RAS*-mutant group and 4.0 months (95% CI, 2.4–5.5) in the wild-type group (*p* = 0.865, **Figure 3B**). For *TP53* status, median PFS was 2.4 months (95% CI, 1.4–3.3) in the mutant group and 1.8 months (95% CI, 1.3–2.3) in the wild-type group (*p* = 0.686, **Figure 3C**); median OS was 4.3 months (95% CI, 2.5–6.1) and 4.2 months (95% CI, 2.8–5.6), respectively (*p* = 0.378, **Figure 3D**). No statistically significant differences in survival outcomes were observed based on *RAS* or *TP53* mutation status.

### Safety

Adverse events are detailed in **Table 4**. Among the 93 patients in the present cohort, the most frequently reported adverse events of any grade were anemia (73.1%), fatigue (51.6%), and neutropenia (47.3%). Neutropenia of grade 3 or higher occurred in 36 patients (38.7%), and febrile neutropenia was observed in 7 patients (7.5%). Notably, the incidence of grade 3 or higher anemia (20.4%) was higher compared to previous clinical trials. Gastrointestinal toxicities such as diarrhea (7.5%) and nausea (19.3%) were relatively infrequent and mostly low-grade, whereas any grade of fatigue was more common (51.6%) compared to prior studies. Biliary complications, including adverse events such as cholangitis and hyperbilirubinemia, were observed in 31 patients (33.3%), with 27 patients (29.0%) experiencing events of grade 3 or higher, likely reflecting the underlying anatomical predisposition to biliary obstruction. In this context, sepsis of grade 3 or higher occurred in 14 patients (15.1%), including 4 cases of grade 5 events.

**Table 4:** Adverse events.

Variable	Present study (n=93)		NIFTY (n=88)		NALIRICC (n=48)*	
	Any grade	Grade ≥3	Any grade	Grade ≥3	Any grade	Grade ≥3
Diarrhea	7 (7.5)	1 (1.1)	20 (22.7)	4 (4.5)	27 (56.2)	7 (14.6)
Nausea	18 (19.3)	2 (2.2)	22 (25.0)	5 (5.6)	29 (60.4)	4 (8.3)
Fatigue	48 (51.6)	11 (11.8)	27 (30.7)	11 (12.5)	18 (37.5)	2 (4.2)
Anorexia	23 (24.7)	1 (1.1)	24 (27.3)	1 (1.1)	8 (16.7)	1 (2.1)
Biliary event†	31 (33.3)	27 (29.0)	NR	NR	4 (4.5)	3 (3.4)
Sepsis	14 (15.1)	14 (15.1)	1 (1.1)	1 (1.1)	3 (6.3)	3 (6.3)
Neutropenia‡	44 (47.3)	36 (38.7)	29 (32.9)	21 (23.9)	13 (27.1)	8 (16.7)
Febrile neutropenia	7 (7.5)	7 (7.5)	2 (2.3)	2 (2.3)	0	0
Anemia	68 (73.1)	19 (20.4)	13 (14.8)	8 (9.0)	9 (18.8)	3 (6.3)

NR, not reported. Data are number of patients (%). *Safety analysis in the NALIRICC trial was based on the 48 patients who received treatment, as one patient did not initiate therapy. †Includes liver infection, increased bilirubin and hepatitis. ‡Includes agranulocytosis, febrile neutropenia, granulocytopenia, neutropenia, neutropenic sepsis, decreased neutrophil count, and pancytopenia.

### Treatment dose intensity

**Supplementary Table S2** presents the treatment exposure and RDI among the 71 evaluable patients who received at least two cycles of chemotherapy. The median treatment duration was 2.1 months (IQR, 1.4–2.3) and was comparable between patients treated in the second-line and third-line or later settings. The median number of treatment cycles was 4 overall, with a slightly lower median in the third-line group (3 cycles) compared to the second-line group (4 cycles). The overall median RDI was 0.69 (IQR, 0.60–0.76), with similar values across subgroups. Only 17 patients (23.9%) achieved an RDI ≥80%, with a higher proportion observed in the second-line group (26.4%) compared to the third-line group (16.7%).

## Discussion

To our knowledge, this is the first real-world, multicenter analysis to evaluate the effectiveness and safety of Nal-IRI/FL in patients with advanced BTC refractory to gemcitabine-based therapy, complementing findings from prior prospective trials. Survival outcomes in this real-world cohort were generally less favorable than those reported in prior prospective trials, likely reflecting the inclusion of a higher proportion of patients with poor performance status and early clinical deterioration. A higher disease burden, particularly the presence of three or more metastatic lesions, was independently associated with inferior PFS and OS. Nevertheless, clinical outcomes were comparable between patients treated in the second- and third-line settings, and treatment efficacy was not significantly influenced by *RAS* or *TP53* mutation status. Notably, hematologic toxicities were more frequent than previously reported in clinical trials, underscoring the importance of careful patient selection when considering Nal-IRI/FL in this setting.

Although the NIFTY and NALIRICC trials reported inconsistent survival outcomes, a recently published pooled analysis of both studies provided more robust evidence supporting the efficacy of Nal-IRI/FL over FL alone (12). This pooled analysis demonstrated significant improvements in median PFS (3.6 months [95% CI, 2.7–4.4] vs. 1.8 months [1.5–2.6]; HR, 0.65 [0.51–0.84]; *p* < 0.001) and a numerical, albeit borderline, improvement in median OS (8.1 months [95% CI, 6.0–8.9] vs. 6.1 months [5.3–7.5]; HR, 0.77 [0.59–1.00]; *p* = 0.051) in favor of Nal-IRI/FL. Compared with prospective trials, our cohort included a higher proportion of patients with ECOG performance status 2 (20.4%), and treatment discontinuation was more frequently observed as a result of adverse events (22.6%) or early clinical deterioration (17.2%). These characteristics of real-world populations likely contributed to the observed inferior survival outcomes in our cohort. Importantly, even among the 76 patients evaluable for radiologic response, survival outcomes remained shorter than those reported in clinical trials, suggesting that the effectiveness of Nal-IRI/FL may be more limited in clinical practice than previously expected.

In the multivariable analysis, a higher disease burden, particularly the presence of three or more metastatic lesions, was independently associated with poorer PFS and OS. This finding is consistent with results from the updated analysis of the NIFTY trial, which also demonstrated that a higher disease burden was linked to shorter PFS and OS (10). Tumor burden, such as the number of metastatic sites, appears to serve as a prognostic factor for survival rather than a predictive marker of treatment response in this setting. Treatment outcomes with Nal-IRI/FL were comparable between patients treated in the second-line and third-line settings. Furthermore, survival outcomes were not significantly affected by fluorouracil resistance. These findings support the consideration of Nal-IRI/FL as a viable subsequent therapeutic option for patients with preserved performance status, even after failure of second-line FOLFOX.

With respect to the impact of molecular alterations on treatment efficacy, the presence of *RAS* or *TP53* mutations did not lead to differences in clinical outcomes in patients treated with Nal-IRI/FL. Although these mutations, especially when co-occurring, are generally linked to a more aggressive tumor phenotype and poorer prognosis in patients with biliary tract cancer (13–15), our findings did not reveal significant differences in PFS or OS based on mutation status. Additionally, patients with *RAS* mutations exhibited a numerically longer PFS, albeit without statistical significance. These findings suggest that Nal-IRI/FL may be considered as a therapeutic option for patients with BTC harboring *RAS* or *TP53* mutations, despite their traditionally poor prognosis. Notably, the numerically longer PFS observed in *RAS*-mutant patients may serve as an exploratory signal that merits further investigation in future studies.

Given the frequent occurrence of hematologic toxicities, careful patient selection remains essential when considering Nal-IRI/FL treatment in real-world settings. Febrile neutropenia occurred in 7 patients (7.5%), and treatment-related death due to grade 5 sepsis was reported in 4 patients (4.3%), underscoring the need for particular caution in patients at high risk for systemic infections. While hematologic toxicities were frequently observed, gastrointestinal adverse events were comparatively less common. This pattern aligns with findings from the recently published pooled analysis of the NIFTY and NALIRICC trials, which highlighted potential ethnic differences in the toxicity profile of liposomal irinotecan (12). Pharmacokinetic variations may underlie these differences, with East Asians more vulnerable to hematologic toxicity and Caucasians to gastrointestinal toxicity. Consistent with these observations, gastrointestinal toxicities were less frequently observed in our cohort.

In our study, approximately one-third of patients experienced biliary complications, most of which were severe. These events likely reflect the characteristics of patients typically encountered in real-world clinical settings, who may not meet the strict eligibility criteria of clinical trials. In such patients, the presence of biliary obstruction or infection may increase the risk of neutropenia-related sepsis, indicating that Nal-IRI/FL should be used with caution in this population.

Regarding dose intensity, the median RDI was relatively low at 0.69, with only one-quarter of patients maintaining an RDI ≥80%. This reduced RDI was primarily attributable to hematologic toxicities, especially neutropenia. As inadequate RDI may compromise the expected antitumor efficacy, appropriate supportive care is essential to ensure adequate dose delivery. In East Asian populations, prophylactic pegylated granulocyte colony-stimulating factor may aid in maintaining dose intensity by mitigating hematologic toxicity, whereas in Western populations, managing gastrointestinal events may be more critical to preserving dose intensity.

This study has several limitations. Early treatment discontinuation, often resulting from rapid clinical deterioration or adverse events, may have hindered the adequate evaluation of chemotherapy efficacy and affected overall survival outcomes. As dose and schedule adjustments were made at the discretion of treating physicians rather than according to a standardized protocol, the resulting suboptimal RDI may have led to an underestimation of the therapeutic efficacy of Nal-IRI/FL. Furthermore, as the study was conducted in a single country with an entirely East Asian population, the generalizability of these findings to broader, more diverse populations may be limited. In addition, this study did not collect data on patient-reported outcomes such as quality of life or symptom burden, which are particularly relevant in the context of later-line treatment for advanced BTCs.

## Conclusions

In this real-world study, Nal-IRI/FL demonstrated modest effectiveness in patients with advanced BTCs refractory to gemcitabine-based therapy. Despite less favorable outcomes compared to clinical trials, its efficacy was consistent across treatment lines and in patients with *RAS* or *TP53* mutations. Given the frequent hematologic toxicities, careful patient selection and supportive care critical to optimizing treatment outcomes. These findings highlight the need for further studies to validate the role of Nal-IRI/FL in more diverse and representative patient populations.

## Data Availability

The datasets presented in this study can be found in online repositories. The names of the repository/repositories and accession number(s) can be found in the article/**Supplementary Material**.

## Supplementary material

Supplementary Table 1 Distribution of genetic alterations in patients with advanced biliary tract cancer. *MAPK* Mitogen-Activated Protein Kinase; *RTK* Receptor Tyrosine Kinase; *DDR* DNA Damage Repair. Data are number of patients (%). *Refers to patients for whom next-generation sequencing testing was conducted.

Supplementary Table 2 Treatment exposure and relative dose intensity according to line of therapy. *RDI* relative dose intensity. Data are n (%) or median (IQR). *Refers to patients who received at least two cycles of chemotherapy.
