# Supplementary material for: Liposomal irinotecan with fluorouracil and leucovorin as salvage treatment for advanced biliary tract cancer refractory to gemcitabine and cisplatin
Source: Front Oncol. 2025 Aug 13;15:1638606. doi: 10.3389/fonc.2025.1638606 (PMC12381770; doi:10.3389/fonc.2025.1638606)
Supplement: Supplementary Table 1 — Distribution of genetic alterations in patients with advanced biliary tract cancer. MAPK Mitogen-Activated Protein Kinase; RTK Receptor Tyrosine Kinase; DDR DNA Damage Repair. Data are number of patients (%). *Refers to patients for whom next-generation sequencing testing was conducted. [file Table1.docx]

**Table S1. Distribution of genetic alterations in patients with advanced biliary tract cancer**

| **Genetic alterations** | **Total* (n=71)** |
| --- | --- |
| **MAPK pathway alterations**  *KRAS* mutation  *NRAS* mutation  *BRAF* mutation | 19 (26.7)  2 (2.8)  2 (2.8) |
| **Cell cycle pathway alterations**  *TP53* mutation  *CDKN2A*/*CDKN1A* mutation | 36 (50.7)  10 (14.1) |
| **RTK pathway alterations**  *FGFR2* fusion/rearrangement  *ERBB2* amplification  *ERBB2*/*3* mutation | 2 (2.8)  2 (2.8)  5 (7.0) |
| **DDR pathway alterations**  *BRCA1*/*2* mutation | 3 (4.2) |
| **IDH1/2 mutation** | 3 (4.2) |
| **Other alterations**  *ARID1A*/*2* mutation  *PIK3CA* mutation | 10 (14.1)  2 (2.8) |
| **No alteration detected** | 6 (8.4) |

*MAPK* Mitogen-Activated Protein Kinase; *RTK* Receptor Tyrosine Kinase; *DDR* DNA Damage Repair. Data are number of patients (%). *Refers to patients for whom next-generation sequencing testing was conducted.

**Table S2. Treatment exposure and relative dose intensity according to line of therapy**

|  | **Total***  **(n=71)** | **2^nd^ line**  **(n=53)** | **≥ 3^rd^ line**  **(n=18)** |
| --- | --- | --- | --- |
| **Median duration of treatment, months** | 2.1 (1.4–3.8) | 2.1 (1.4–4.4) | 2.0 (1.3–2.9) |
| **Median cycles of treatment** | 4 (2–6) | 4 (2.5–6.5) | 3 (2.0–4.2) |
| **Median relative dose intensity** | 0.69 (0.60–0.76) | 0.69 (0.60–0.78) | 0.68 (0.58–0.72) |
| **RDI ≥ 80%** | 17 (23.9) | 14 (26.4) | 3 (16.7) |

*RDI* relative dose intensity. Data are n (%) or median (IQR). *Refers to patients who received at least two cycles of chemotherapy.
